# Nuclear β-catenin accumulation is associated with increased expression of Nanog protein and predicts poor prognosis of non-small cell lung cancer

**DOI:** 10.1186/1479-5876-11-114

**Published:** 2013-05-06

**Authors:** Xi-Qing Li, Xing-Long Yang, Gong Zhang, Si-Pei Wu, Xu-Bing Deng, Sheng-Jun Xiao, Qiu-Zhen Liu, Kai-Tai Yao, Guang-Hui Xiao

**Affiliations:** 1 Cancer Institute, Southern Medical University, 1023 Shatai Road South, Guangzhou 510515, China; 2 Hospital of Guilin Medical University, Guilin, China

**Keywords:** β-catenin, Nanog, Immunohistochemistry, Prognosis, Lung cancer

## Abstract

**Background:**

Although the prognostic roles of β-catenin expression in non-small cell lung cancer (NSCLC) have been reported in several immunohistochemical (IHC) studies, the results were not consistent because some studies lack sufficient number of the positive cases or did not evaluate the subcellular localization features of the protein.

**Method:**

In this study, we have evaluated the expression levels and subcellular localization of β-catenin and Nanog proteins IHC staining in tissue specimens from 309 patients with NSCLC, and explored their association with clinicopathological features and patient outcome.

**Results:**

We showed that patients with negative expression of membranous beta-catenin had a trend towards shorter survival (*p*=0.064) than those with positive expression. In contrast to previous studies, we found that increased expression of either cytoplasmic or nuclear β-catenin was strongly associated with poor prognosis and was an independent prognosticator for overall survival (*p* <0.01). We further found that NSCLC cells frequently exhibited an abundance of nuclear Nanog protein which was significantly correlated with nuclear β-catenin expression (*p* <0.01) and poor prognosis (*p* <0.01). Interestingly, immunofluorescent staining results revealed that increased expression of Nanog and nuclear translocation of β-catenin occurred concomitantly in response to epidermal growth factor receptor(EGFR) signaling in A549 and H23 cells. Furthermore, western blot analysis show that nuclear β-catenin rather than cytoplasmic β-catenin expression in the A549 and H23 cells can be enhanced by adding EGF, Nanog expression in the A549 and H23 cells with knockdown of β-catenin can not be obviously enhanced by adding EGF.

**Conclusion:**

We propose that evaluation of subcellular localization of β-catenin and Nanog expression is of clinical significance for patients with NSCLC.

## Introduction

Lung cancer is the most common cancer worldwide, and has the highest incidence and mortality levels of any cancer [1]. NSCLC is thought to originate in lung epithelial cells, and comprises diverse histological subtypes including adenocarcinoma, bronchioloalveolar, squamous, anaplastic and large-cell carcinomas, and approximately 85% of lung cancers are identified as NSCLC [2]. Advances in surgical, radiotherapeutic, and chemotherapeutic approaches have been made, but the long-term survival rate remains low [3], with a 5-year overall survival of 9-15% and a median survival time of 16 to 18 months [4]. The aggressive and heterogeneous nature of lung cancer has thwarted efforts to reduce mortality from the NSCLC. Thus, there is an urgent need for the determination of useful prognostic molecular markers for clinical management of patients with NSCLC.

β-catenin is a key component of the canonical Wnt pathway that plays pivotal roles in pattern formation during embryogenesis and in malignant transformation [5]. At the plasma membrane, β-catenin is associated with the cadherin class of cell-adhesion proteins and functions in regulating cell adhesion [6]. In the absence of Wnt signal, cytoplasmic β-catenin interacts with glycogen synthase kinase-3β (GSK-3β) in a large complex known as destruction complex which also contains the adenomatous polyposis coli (APC) and the axis inhibition protein (Axin). Phosphorylation of β-catenin at its N-terminus by GSK-3β leads to its degradation via the ubiquitin/proteasome pathway. When Wnt pathway is activated, β-catenin phosphorylation by GSK-3β is inhibited. The hypophosphorylated β-catenin is stabilized and translocated to the nucleus, where it binds T-cell factor/lymphoid enhancer factor (TCF/Lef) and activates its target gene expression [7].

Nanog is a core transcription factor required for maintenance of the pluripotency and self-renewal of embryonic stem cells. Nanog protein has been identified as one of four factors essential for reprogramming adult cells into induced pluripotent stem (iPS) cells [8]. Recent studies have revealed that Nanog is also involved in self-renewal and tumorigenicity of cancer stem cells in a variety of human cancers [9-12]. Increased expression of Nanog protein was observed in a panel of specimens of lung adenocarcinoma and ectopic coexpression of Nanog and another stem cell factor Oct4 enhanced tumor-initiating ability, epithelial–mesenchymal transition, and tumorigenesis in a lung cancer cell line [13].

Nanog is critically involved in regulation of cancer stem cells in several types of tumors [9-12] and has been reported to be target gene of β-catenin that inhibits differentiation by increasing the expression of Nanog [14]. We also demonstrated Nanog expression could be influenced by β-catenin in nasopharyngeal carcinoma in our earlier study [15].

In this study, we have evaluated the expression levels and subcellular localization of β-catenin and Nanog proteins in primary tumor specimens from 309 patients with NSCLC, and explored their association with clinicopathological features and patient outcome.

## Materials and methods

### Patients and tumor specimens

Primary tumor specimens from 309 NSCLC patients who underwent curative surgical resection were evaluated in this study. None of these patients had undergone chemotherapy or radiotherapy prior to the surgery. Of these patients, 203 were collected from 2001 to 2005 at Hunan Tumor Hospital and the rest of 96 were from 2002 to 2006 at the Affiliated Hospital of Guilin Medicine University. Patients in the study were examined and treated according to provincial guidelines. This study was approved by the Research Ethics Boards of the Southern Medical University, China.

Cores from formalin-fixed paraffin embedded tumor tissues of NSCLC were arrayed in triplicate onto a tissue microarray (TMA) and each TMA block consisted of up to 40 tissue cores, before the area of cores were chosen, the preliminary experiment of β-catenin were made to ensure the typical cores. TMAs were constructed using an automated tissue arrayer (Beecher Instruments Inc. USA) by Auragene Bioscience Corporation, China. Tissues core with a diameter of 3 mm was transferred to a recipient paraffin block (array margin of 15mm×20 mm). The block was sectioned at a series of 4-μm-thick slices. Once the TMA slices were made, they were heat 60°C for 1 h to aid cutting.

Relevant clinical pathologic features (Table 1) were obtained from the patients’ files and/or by telephone interviews with the patients or their relatives. Pathological stages were classified according to the TNM staging system. Histological grading and typing of the tumor were determined according to the World Health Organization tumor classification system.

**Table 1:** **Correlation between the clinicopathologic characteristics and membranous**, **cytoplasmic and nuclear expression of β**-**catenin in 316 NSCLC patients**

		**MEMBRANOUS(n, %)**			**CYTOPLASMIC(n, %)**			**NUCLEAR(n, %)**		
Variables	Cases	Low(102)	High(207)	*p*^*a*^	Low(176)	High(133)	*p*^*a*^	Low(170)	High(139)	*p*^*a*^
Gender										
Male	134	42(31.3)	92(68.7)	0.586	74(55.2)	60(44.8)	0.590	73(54.5)	61(45.5)	0.868
Female	175	60(34.3)	115(65.7)		102(58.3)	73(41.7)		97(55.4)	78(44.6)	
Age (years)^*b*^										
<52	149	59(39.6)	90(60.4)	0.017	85(57.0)	64(43.0)	0.976	77(51.7)	72(48.3)	0.255
≥52	160	43(26.9)	117(73.1)		91(56.9)	69(43.1)		93(58.1)	67(41.9)	
Histologic subtype										
Squamous carcinoma	142	47(33.1)	95(66.9)	0.976	87(61.3)	55(38.7)	0.158	85(59.9)	57(40.1)	0.115
Adenocarcinoma	167	55(32.9)	119(67.1)		89(53.3)	78(46.7)		85(50.9)	82(49.1)	
Histologicalgrade										
Grade 1	126	34(27.0)	92(73.0)	0.032	75(59.5)	51(40.5)	0.644	83(65.9)	43(34.1)	0.001
Grade 2	112	36(32.1)	76(67.9)		60(53.6)	52(46.4)		59(52.7)	53(47.3)	
Grade 3	71	32(45.1)	39(54.9)		41(57.7)	30(42.3)		28(39.4)	43(60.6)	
**T classification**										
**T1**	**71**	**23**(**32**.**4**)	**48**(**67**.**6**)	**0**.**069**	**43**(**60**.**6**)	**28**(**39**.**4**)	**0**.**349**	**40**(**56**.**3**)	**31**(**43**.**7**)	**0**.**727**
**T2**	**58**	**23**(**39**.**7**)	**35**(**60**.**3**)		**37**(**63**.**8**)	**21**(**36**.**2**)		**32**(**55**.**2**)	**26**(**44**.**8**)	
**T3**	**126**	**39**(**31**.**0**)	**87**(**69**.**0**)		**70**(**55**.**6**)	**56**(**44**.**4**)		**72**(**57**.**1**)	**54**(**42**.**9**)	
**T4**	**54**	**17**(**31**.**5**)	**37**(**68**.**5**)		**26**(**48**.**1**)	**28**(**51**.**9**)		**26**(**48**.**1**)	**28**(**51**.**9**)	
**Clinical stage**										
**I**	**108**	**35**(**32**.**4**)	**73**(**67**.**6**)	**0**.**818**	**62**(**57**.**4**)	**46**(**42**.**6**)	**0**.**957**	**56**(**51**.**9**)	**52**(**48**.**1**)	**0**.**689**
**II**	**81**	**29**(**35**.**8**)	**52**(**64**.**2**)		**45**(**55**.**6**)	**36**(**44**.**4**)		**45**(**55**.**6**)	**36**(**44**.**4**)	
**III**	**120**	**38**(**31**.**7**)	**82**(**68**.**3**)		**69**(**57**.**5**)	**51**(**42**.**5**)		**69**(**57**.**5**)	**51**(**42**.**5**)	

^a ^Chi-square test.

^b ^Patients were divided according to the median values of age.

### Immunohistochemistry (IHC) staining

Serial tumor sections and IHC staining were used to evaluate both β-catenin and Nanog proteins. β-catenin immunoactivity was examined using a mouse polyclonal antibody (Cell Signaling Technology, Danvers). Nanog protein was detected with a rabbit polyclonal antibody (Cell Signaling Technology, Danvers). The TMA blocks containing tissue sections (4 μm) were de-waxed in xylene, rehydrated through a graded series of ethanol solutions, rinsed in distilled water for 5 min, and then immersed in boiling citrate buffer in methanol for 1.5 min for antigen retrieval. After deparaffinization and rehydration, blocks were incubated in 3% hydrogen peroxide to block endogenous peroxidase activity. Then the sections were subject to immunohistochemistry staining using the primary antibodies overnight at 4°C in a humidity chamber. The avidin-biotin technique was applied using DAB for visualization and hematoxylin for nuclear counterstaining. Negative controls were prepared by omitting the primary antibody. Histological and IHC evaluation were independently performed by two pathologists without knowledge of the clinicopathological outcomes of the patients. We classified the IHC staining results into three categories according to subcellular localization of β-catenin, e.g. membranous, cytoplasmic and nuclear. Briefly, each slide was examined in its entirety under a light microscope, and an initial score was assigned which represented the estimated proportion of positive tumor cells (0: ≤5%; 1: 5~25%; 2: 25~75%; 3: ≥75%). The score 0 was defined as low and 1, 2, 3 as high. Slides with indeterminate evaluation were re-evaluated, and a consensus was reached.

### Immunofluorescent staining

For immunofluorescent staining, A549 [16] or H23 [17] cells grown on the surface of cover slides were serum-starved overnight followed by stimulation with 50 ng/ml EGF for 24 hours. Cells were fixed with 4% paraformaldehyde, rehydrated in PBS, and incubated with mouse anti-β-catenin (Santa Cruz Biotech) and rabbit anti-Nanog primary antibodies (Cell Signaling Technology, Danvers) at room temperature for 40 min. Subsequently, cells were incubated with Alexa Fluor 488-conjugated anti-rabbit antibody and Alexa Fluor 594-conjugated anti-mouse antibody (Molecular Probes; Invitrogen Corp.) for 40 min at room temperature. The nuclei were stained with DAPI. Slides were examined with a fluorescent confocal microscope (Olympus FV1000, Japan).

### Western blot analysis

Cytoplasmic and nuclear proteins of A549 and H23 cells were extracted by Nuclear and Cytoplasmic Protein Extraction Kit (Beyotime), then equal amount of protein were subjected to electrophoresis on a SDS-PAGE gel. The separated proteins were transferred to PVDF membranes (Millipore) and probed with appropriate primary antibodies. Protein bands were detected by enhanced chemiluminescence reagents (Pierce Biotechnology).

### Lentiviral constructs and infection of NSCLC cells

Both pLKO.1 lentiviral shRNA vector and control shRNA targeting against GFP were from Aldrich -Sigma. β-catenin targeting sequences are GCTTGGAATGAGACTGCTGAT, which was previously described in our earlier study [15]. The sense and antisense oligonucleotides were annealed and ligated into pLKO.1 lentiviral vector. The viruses were packaged in 293T cells according to standard protocols. Viral production and infection of target cells were previously described [18]. Infected cells were selected with 2 μg/mL puromycin.

### Statistical methods

Statistical analysis was performed using the SPSS 13.0 software package for Windows. Associations between clinicopathological features and IHC β-catenin and Nanog expression were analyzed using the chi-squared test. Multivariate survival analyses were performed with the Cox regression model. Overall survival (OS) was measured from the onset of treatment to the date of death or the survival status at the last date of follow-up. OS probabilities were estimated by the Kaplan-Meier method and the significance of differences was assessed by the log-rank test. Correlations between β-catenin and Nanog expression with clinicopathological factors were analyzed using Fisher’s exact probability test or the chi-squared test. A *p*-value < 0.05 was considered statistically significant.

## Results

### Clinicopathological features

Tumor specimen from 309 NSCLC patients consisting of 134 males and 175 female were included in this study. The median age was 52 years (range: 37–82 years). Of these patients, 167 (32.9%) were diagnosed as adenocarcinoma and 142 (33.1%) diagnosed as squamous carcinoma. Clinical stages, histological grades, and studied proteins were correlated as shown in Table 1. The tumor tissues was classified in three histological grades: grade 1 (126 patients), grade 2 (112 patients) and grade 3 (71 patients). The expression of membranous and nuclear, but not cytoplasmic, β-catenin expression were correlated with the histological grade (p<0.05), as shown in Table 1. Nanog expression that was localized in the nucleus was also correlated with the histological grade (*p* <0.01, Table 2).

**Table 2:** The clinicopathologic characteristics of NANOG expression in 316 NSCLC patients

			**NANOG(n, %)**	
Variables	case	Low(215)	High(94)	*p*^*a*^
Gender				
Male	134	90(67.2)	44(32.8)	0.419
Female	175	125(71.4)	50(28.6)	
Age (years)^*b*^				
<52	149	99(66.4)	50(33.6)	0.248
≥52	160	116(72.5)	44(27.5)	
Histologic subtype				
Squamous carcinoma	142	103(72.5)	39(27.5)	0.298
Adenocarcinoma	167	112(67.1)	55(32.9)	
Histologicalgrade				
Grade 1	126	105(83.3)	21(16.7)	<0.001
Grade 2	112	80(71.4)	32(28.6)	
Grade 3	71	30(42.3)	41(57.7)	
**T classification**				
**T1**	**71**	**50**(**70**.**4**)	**21**(**29**.**6**)	**0**.**572**
**T2**	**58**	**36**(**62**.**1**)	**22**(**37**.**9**)	
**T3**	**126**	**91**(**72**.**2**)	**35**(**27**.**8**)	
**T4**	**54**	**38**(**70**.**4**)	**16**(**29**.**6**)	
**Clinical stage**				**0**.**358**
**I**	**108**	**75**(**69**.**4**)	**33**(**30**.**6**)	
**II**	**81**	**61**(**75**.**3**)	**20**(**24**.**7**)	
**III**	**120**	**79**(**65**.**8**)	**41**(**34**.**2**)	

^a ^Chi-square test.

^b ^Patients were divided according to the median values of age.

### Expression of β-catenin in NSCLC

**Figure 1:**
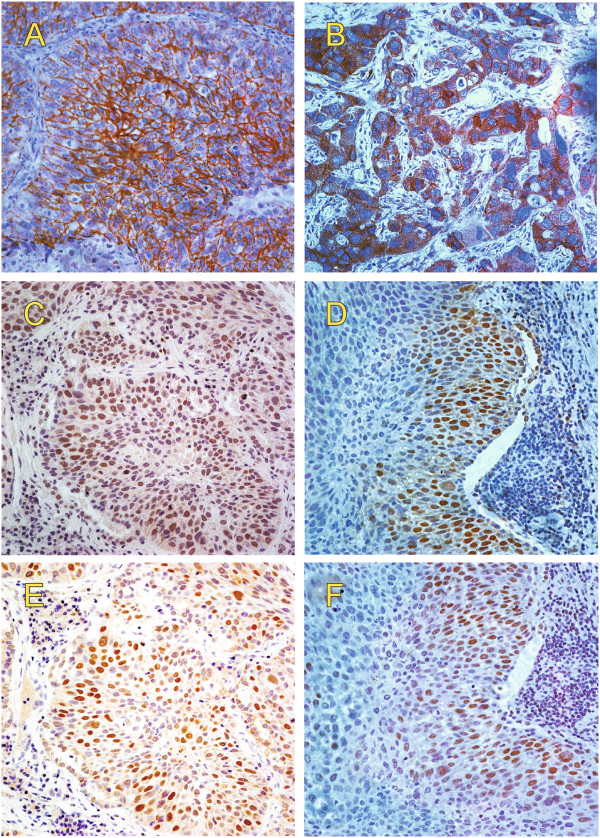
**IHC staining for β**-**catenin and Nanog expression in NSCLC.** Representative images of intracellular β-catenin expressed at the membrane (**A**), in cytoplasma (**B**) or in the nucleus (**C **and **D**). In serial sections, Nanog staining (**E **and **F**) frequently overlapped with β-catenin staining (**C **and **D**) in the nuclei of most cells.

Representative images of β-catenin immunohistochemical staining in NSCLC tissues are shown in Figure 1A-C. Positive membranous, cytoplasmic, and nuclear expression of β-catenin was detected in 67.0% (207/309), 43.0% (133/309), and 45.0% (139/309) of the NSCLC tissues, respectively.

Nanog is critically involved in regulation of cancer stem cells in several types of tumors and has been reported to be transcriptionally regulated by β-catenin. Thus we further examined Nanog expression in NSCLC specimen. We found that 30.4% (94/309) tumor tissues displayed Nanog immunoactivity that was located in the nucleus in most cases (Figure 1D).

### Follow-up outcome

**Figure 2:**
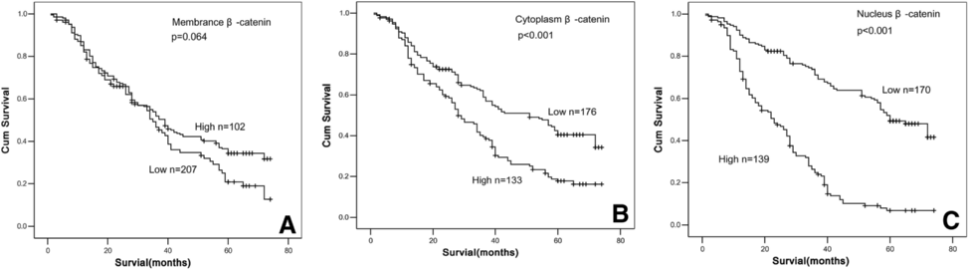
**The Kaplan**-**Meier analysis of survival rate of patients according to status of β**-**catenin expression. **The influence of membranous (**A**), cytoplasmic (**B**), and nuclear (**C**) β-catenin expression on the prognosis of patients with NSCLC is shown.

**Figure 3:**
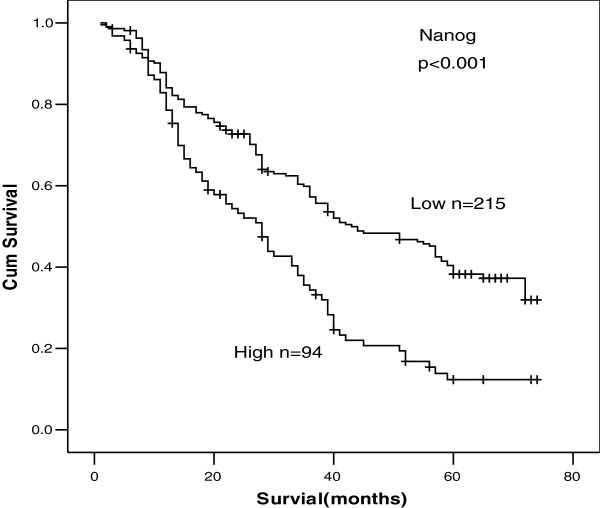
**The Kaplan**-**Meier analysis of survival rate of patients according to status of Nanog expression.**

The last follow-up date is Sep. 29th, 2009, with a median follow-up time 52 months (range 7–69.5 months). The 1-, 3- and 5-year overall survival (OS) rates were 82.4%, 52.5%, 30.6%, respectively. The survival rates of 309 NSCLC patients according to status of β-catenin and Nanog were shown in Table 2. The survival rate of patients with nuclear β-catenin expression was significantly lower than that of patients without nuclear β-catenin expression (*p* <0.01, Figure 2C). Likewise, the survival rate of patients with cytoplasmic β-catenin expression was significantly lower than that of patients without cytoplasmic β-catenin expression (*p* <0.01, Figure 2B). However, there was no statistical difference in the survival rate between patients with or without membranous β-catenin (*p*=0.064, Figure 2A), but with a trend toward reduced survival in patients with loss of β-catenin expression. Patients without Nanog protein expression had a significantly better prognosis and longer survival. The overall survival rate was 12.3% (Nanog-positive) and 38.3% (Nanog-negative) for this group (*p* <0.01) (Figure 3).

Univariate analyses showed no significant association between OS and age (>52 yr vs. ≤52 yr), sex (female vs. male), histological subtype (adenocarcinoma vs. squamous carcinoma), T stage (T3-T4 vs. T1-T2), clinical stage (stage III vs. I-II) (Table 3). The expression levels of cytoplasmic and nuclear β-catenin and Nanog were an independent prognosticator for OS (Table 3). In a multivariate analysis incorporating all clinicopathologic variables and covariates as shown in Table 3, comparisons between genders ages, tumor grades, tumor T stages, tumor C stages, expression levels of Nanog protein (low vs. high), and intracellular β-catenin protein expression at the membrane, in cytoplasm and in the nucleus (low vs. high) were performed to identify independent prognostic factors.

**Table 3:** Univariate and multivariate analysis for clinicopathologic variables

	**Univariate analysis**			**Multivariate analysis**		
**Parameters**	**HR**^***a***^	**95% CI**^***b***^	***P***^***c***^	**HR**^***a***^	**95% CI**^***b***^	***P***^***c***^
Gender						
Male vs. female	1.032	0.782-1.363	0.826			
Age (years)						
<52 vs.≥52	0.995	0.756-1.311	0.948			
Histologic subtype						
ad vs l	1.111	0.842-1.466	0.456			
T classification						
T1–T2 vs. T3–T4	0.956	0.832-1.099	0.529			
**C classification**						
**I**-**II vs**. **III**	0.961	0.818-1.129	0.629			
Differention						
Grade1,Grade2,Grade3	1.520	1.272-1.816	0.000	**1**.**526**	**1**.**273**-**1**.**829**	**0**.**000**
Membranous						
positive and negative	0.763	0.571-1.021	0.069	0.820	0.609-1.106	0.193
Cytoplasmic						
positive and negative	1.815	1.375-2.396	0.000	1.871	1.412-2.478	0.000
Nuclear						
positive and negative	3.864	2.872-5.200	0.000	3.776	2.786-5.118	0.000
Nanog						
positive and negative	1.994	1.494-2.661	0.000	1.700	1.249-2.315	0.001

^a ^HR Harzard Rate.

^b ^CI Confidence Interval.

^c ^Cox regression model.

The expression levels of cytoplasmic and nuclear β-catenin and Nanog were an independent prognosticator for overall survive.

### Correlation between β-catenin and Nanog expression in NSCLC

**Figure 4:**
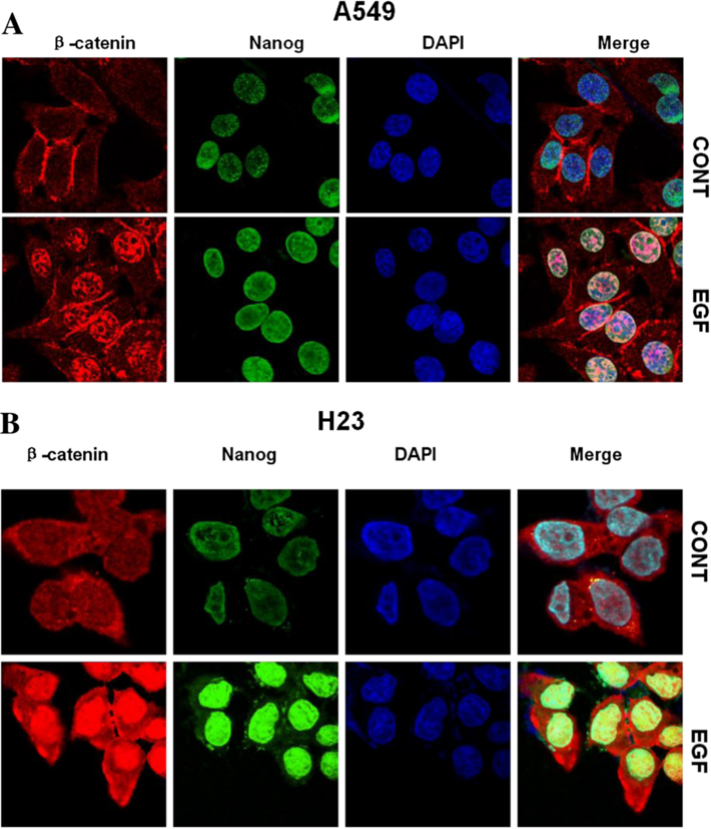
**Expression of β**-**catenin and Nanog was concomitantly regulated by EGFR signaling. **A549 (**A**) and H23 (**B**) cells were treated without or with EGF followed by immunofluorescent staining. In the absence of EGF, β-catenin was located predominantly at the plasma membrane, with faint staining distributed in the cytoplasm. Faint Nanog staining was detected in the nucleus. When cells were treated with EGF, β-catenin staining translocated to the nucleus in both cell lines and nuclear Nanog staining was moderately increased in A549 cells and abundantly accumulated in H23 cells.

We further analyzed correlation between expression status of Nanog and β-catenin. As mentioned above, positive Nanog protein staining was almost exclusively observed in nucleus and β-catenin expression was stratified according to its subcellular locations (Table 4). Seventy two of the 139 (51.8%) tumor tissues that stained positive for nuclear β-catenin also displayed Nanog immunoactivity. Among the 170 specimen without nuclear β-catenin expression, only 22 (12.94%) showed Nanog immunoreactions (Fig. 4C, *p* < 0.001). On the contrary, only 55 (26.6%) of the 207 specimen with membranous β-catenin expression showed Nanog immunoreactions (Table 4, *p* = 0.036). Likewise, cytoplasmic β-catenin expression is not correlated with Nanog protein (Table 4, *p* = 0.166) even though cytoplasmic β-catenin is associated with poor prognosis. However, expression of cytoplasmic and nuclear β-catenin is correlated, among the 133 specimen with cytoplasmic β-catenin expression, 105 (78.9%) showed nuclear β-catenin immunoreactions (Table 5, p < 0.001).

**Table 4:** **Correlation between β**-**catenin and Nanog expression**

			**Nanog**		***p***^***a***^
			**-**	**+**	
Membranous	**102**	-	**63**(**61**.**8**)	**39**(**38**.**2**)	**0**.**036**
	**207**	+	**152**(**73**.**4**)	**55**(**26**.**6**)	
Cytoplasmic	**176**	-	**128**(**72**.**7**)	**48**(**27**.**3**)	**0**.**166**
	**133**	+	**87**(**65**.**4**)	**46**(**34**.**6**)	
Nuclear	**170**	-	**148**(**87**.**1**)	**22**(**12**.**9**)	<**0**.**001**
	**139**	+	**67**(**48**.**2**)	**72**(**51**.**8**)	

^a ^Chi-square test.

Nanog is significantly associated with membranous or nuclear β-catenin expression (*p* <0.05). No statistical correlation exists between cytoplasmic β-catenin and Nanog proteins(*p*=0.166).

**Table 5:** **Correlation between cytoplasmic and nuclear of β**-**catenin expression**

			**Nuclear**		***p***^***a***^
			**-**	**+**	
**Cytoplasmic**	**176**	-	**142**(**80**.**7**)	**34**(**19**.**3**)	<**0**.**001**
	**133**	+	**28**(**21**.**1**)	**105**(**78**.**9**)	

^a ^Chi-square test.

Cytoplasmic β-catenin is significantly associated with Nuclear β-catenin expression (p <0.001).

### Expression of β-catenin and Nanog was concomitantly regulated by EGFR signaling

EGFR signal transduction pathway is critically involved in cell proliferation and survival of tumors of epithelial cell origin [19]. Increased expression of EGFR has been detected in 40%–80% of NSCLC and has been associated with advanced disease and poor prognosis [20]. Recently, we demonstrated that activation of EGFR increased cancer stem-like cell properties in nasopharyngeal carcinoma, and this effect of EGFR was mediated by PI3K/AKT/β-catenin signaling [15]. In the present study, high incident of nuclear β-catenin was detected in lung cancer specimens and was associated with poor survival. Thus we asked if EGFR signaling could induce β-catenin activation in NSCLC. For this purpose, serum-starved A549 (Figure 4A) or H23 (Figure 4B) cells were incubated without or with EGF and subjected to immunofluorescent staining. In the absence of EGF, β-catenin was located predominantly at the plasma membrane, with faint staining distributed in the cytoplasm. When cells were treated with EGF, β-catenin staining translocated to the nucleus in both A549 and H23 cells. These results suggested that nuclear accumulation of β-catenin can be induced not only by Wnt signaling, but also by EGF stimulation in NSCLC. In addition, nuclear Nanog expression was dramatically enhanced in response to EGFR activation. These results of in vitro experiments with established cell lines are consistent with our finding that nuclear β-catenin is significantly associated with Nanog expression in primary NSCLC specimens.

### Nuclear β-catenin is requirement in the regulation of Nanog

**Figure 5:**
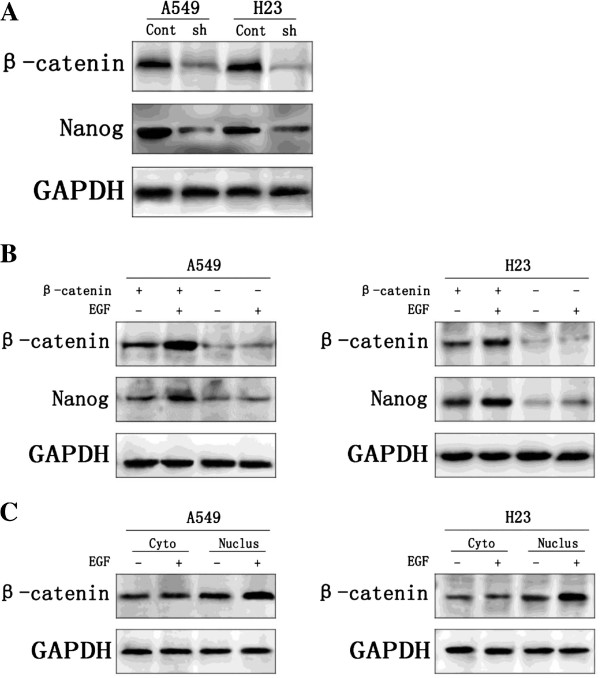
**Nuclear β**-**catenin is requirement in the regulation of Nanog. **A549 cells and H23 cells infected with control shRNA lentivirus(Cont) or β-catenin shRNA(sh). In both cell lines, β-catenin protein was markedly knocked down with β-catenin shRNA. Knockdown of β-catenin also decreased the Nanog expression. All experiments were carried 3 times independently (Figure 5A). Nanog expression in the A549 and H23 cells with knockdown of β-catenin can not be obviously enhanced by adding EGF, but on the other hand, when the expression of β-catenin is increased by adding EGF, Nanog thereupon increased expression upon β-catenin (Figure 5B). Nuclear β-catenin(Nucles) rather than cytoplasm β-catenin(Cyto) expression in the A549 and H23 cells can be enhanced by adding EGF (Figure 5C).

We investigated the requirement of β-catenin in the regulation of Nanog in A549 and H23. To this end, β-catenin was knocked down with lentivirus-mediated shRNAs (Figure 5A). Compared with cells infected with the control shRNA lentivirus, cells infected with β-catenin shRNA lentivirus exhibited reduced Nanog expression. Moreover, Nanog expression in the NSCLC cells with knockdown of β-catenin can not be obviously enhanced by adding EGF, but on the other hand, when the expression of β-catenin is increased by adding EGF, Nanog thereupon increased expression upon β-catenin (Figure 5B). It is worthy of note that nuclear β-catenin rather than cytoplasm β-catenin expression in the NSCLC cells can be enhanced by adding EGF, (Figure 5C).

## Discussion

During the past decade, mounting evidence has well demonstrated that accumulation and nuclear localization is a hallmark of Wnt/β-catenin pathway activation. In addition to controlling cell-cell adhesion through its binding with cadherin at the membrane, β-catenin acts as a transcriptional activator in the nucleus where it interacts with LEF1 and TCF transcription factors and regulates transcription of target genes responsible for cell proliferation and differentiation. Thus, subcellular localization of β-catenin from cell membrane to the nucleus determines its two distinct functions. In agreement with these findings, cytoplasmic and nuclear β-catenin expression has been reported to be associated with a poorer prognosis in patients with cancers of breast, liver, and colon [21-23].

The prognostic roles of β-catenin expression in NSCLC have been extensively studied in the past years [24,25]. In a very recent study reported by Chiu et al. on 370 cases of NSCLC, β-catenin expression was found to be negative in 28% of tumors and positive in 72% of tumors. The lack of β-catenin expression was significantly associated with poor survival and retained independent prognostic significance [26]. Similar results were also observed in a number of early studies. In the present study, we evaluated the expression of β-catenin in 309 tissue specimens of NSCLC. In order to more precisely analyze IHC results in relation to patient prognosis and survival, we have classified the IHC staining results into three categories according to subcellular localization of β-catenin. We showed that patients with negative expression of membranous β-catenin had a trend of shorter survival (*p* = 0.067) than those with positive expression. This result is in agreement with the findings reported by others previously.

In addition to the similarity, there are some striking differences between our results and those of earlier studies. We found that increased expression of nuclear β-catenin was strongly associated with poor prognosis of patients with NSCLCs. In addition, multivariate analysis revealed that the expression of nuclear β-catenin was an independent prognosticator for OS. These data are consistent with recent observations that increased Wnt/β-catenin pathway activity is associated with metastasis and relapse in primary lung adenocarcinoma [27]. Importantly, even though we revealed that cytoplasmic β-catenin was a prognosticator, these observations do not exclude the possibility that nuclear β-catenin acts primarily on the prognosis, since a majority of cases over expressing cytoplasmic β-catenin also contain nuclear β-catenin expression. We further demonstrated by immunofluorescent staining and/or western blot analysis that independent to Wnt, nuclear accumulation of β-catenin can be induced by EGFR pathway that is critically involved in tumorigenesis of NSCLC. Our in vitro results provide not only supportive evidence to our IHC finding that nuclear β-catenin is significantly associated with Nanog expression in primary NSCLC specimens, but also an additional mechanism by which β-catenin activation is regulated by growth factor signaling in a Wnt-independent manner in NSCLC.

In contrast to our investigation, some earlier studies evaluated only the overall intensity of IHC staining of β-catenin in tumor cells without stratification of the data based on subcellular localization of the protein. Thus the distinct impact of β-catenin at different cellular compartments on patient prognosis and survival was ignored. Some of the studies [28,29] evaluated cytoplasmic or nuclear β-catenin specifically, but found no correlation between cytoplasmic or nuclear staining and clinicopathological parameters or survival rates, probably due to low numbers of the positive cases identified in those studies. One of the early study [30] showed that increased expression of cytoplasmic and nuclear β-catenin can predict favorable prognosis of NSCLCs patients. However, β-catenin immunoactivity was found to be associated with increased proliferation as suggested by high Ki-67 expression in the same set of specimens.

Nanog protein is elevated in many human tumors including NSCLC and might positively regulate tumor metastasis through enhancing EMT in lung adenocarcinoma [13]. We found that Nanog is expressed in significant portion of specimens and Nanog immunoactivity was observed in the nucleus. Expression of Nanog is significantly correlated with nuclear, but not membranous nor cytoplasmic, β-catenin. Furthermore, our IHC staining demonstrated that increased expression of Nanog and nuclear translocation of β-catenin occurred concomitantly in response to EGFR signaling in A549 and H23 lung adenocarcinoma cells. This correlation is in agreement with a previous report demonstrating that β-catenin up-regulates Nanog expression in embryonic stem cells [31]. We proposed that Nanog contributes to tumorigenesis and represents an important prognostic marker of poor prognosis in patients with NSCLC.

Taken together, we showed that negative expression of membranous β-catenin correlated with a shorter survival time than the normal expression level of the protein. However, high expression of nuclear β-catenin was strongly associated with poor prognosis and was an independent prognosticator for OS. We further found that NSCLC cells frequently exhibited nuclear Nanog protein abundance that is significantly correlated with nuclear β-catenin expression and poor prognosis. Furthermore, results from IHC staining with established lung cancer cell lines revealed that increased expression of Nanog and nuclear translocation of β-catenin occurred concomitantly in response to EGFR signaling. In conclusion, we propose that evaluation of subcellular localization of β-catenin and Nanog expression is of clinical significance for patients with NSCLC.

## Competing interests

The authors declare no conflict of interest.

## Authors’ contribution

XQL carried out the cases collection, immunohistochemical staining and western blot analysis. XLY and GZ conducted immunofluorescent staining. SPW and XBD performed the statistical analysis. SJX constructed the tissue microarrays. QZL participated in the design of the study. GHX and KTY designed and conceived of the study, XQL helped to draft the manuscript. GHX wrote the manuscript. All authors have read and approved the final manuscript.
